# Fluorinated Poly(*p*-phenylenevinylene)s: Synthesis and Optical Properties of an Intriguing Class of Luminescent Polymers

**DOI:** 10.3390/ma3053077

**Published:** 2010-05-07

**Authors:** Gianluca M. Farinola, Antonio Cardone, Francesco Babudri, Carmela Martinelli, Francesco Naso, Giovanni Bruno, Maria Losurdo

**Affiliations:** 1 Dipartimento di Chimica, Università degli Studi di Bari “Aldo Moro”, Via Orabona 4, Bari I-70125, Italy; 2 Istituto di Chimica dei Composti Organometallici, CNR-ICCOM di Bari, Dipartimento di Chimica, Università degli Studi di Bari “Aldo Moro”, Via Orabona 4, Bari I-70125, Italy; E-Mail: cardone@ba.iccom.cnr.it (A.C.); 3 Istituto per le Metodologie Inorganiche e dei Plasmi, CNR-IMIP di Bari, Dipartimento di Chimica, Università degli Studi di Bari “Aldo Moro”, Via Orabona 4, Bari I-70125, Italy; E-Mail: giovanni.bruno@ba.imip.cnr.it (G.B.)

**Keywords:** poly(*p*-phenylenevinylene)s, conjugated polymers, organic semiconductors, fluorinated polymers, synthesis

## Abstract

This review is an overview of our previous work on the synthesis and properties of poly(*p*-phenylenevinylene)s (PPVs) selectively fluorinated in different positions of the conjugated backbone. Both the synthetic challenges and the effects of functionalization with fluorine atoms on the optical behavior are discussed, highlighting the peculiarities and the interest of this class of conjugated polymers. A general polymerization protocol for PPVs, that is based on the Pd-catalyzed Stille cross-coupling reaction of bis-stannylated vinylene monomers with aromatic bis-halides, has been successfully extended to the synthesis of selectively fluorinated poly(*p*-phenylenevinylene)s. The properties of a series of these PPVs differing in the number and positions of the fluorine atoms on the conjugated backbone have been studied, even in comparison with the non-fluorinated counterparts. The intriguing optical features of the resulting materials are discussed considering not only the role of the electronic and steric effects induced by the fluorine substituents, but also the impact of the fluorination on the solid state organization and intermolecular interactions.

## 1. Introduction

Poly(arylenevinylene)s (PAV)s have been extensively investigated in the last decades, especially for their use as emissive layers in organic light-emitting diodes (OLEDs). Two main drawbacks have limited until now the wide commercial applications of devices based on this class of conjugated polymers. First, the stability of the conjugated backbone, especially the double bonds, against photo-oxidation processes is not adequate to match the lifetime requirements of the displays market. As a second issue, the overly high LUMO energy makes PPV polymers poor electron acceptors. This requires the use of low work function metals, such as calcium, as cathode in OLED devices to make the electron injection more efficient. However, these metals suffer from high susceptibility to atmospheric degradation, while more stable higher work function metals, such as aluminum, are attractive especially for commercial applications. As predicted by theoretical calculations and shown by the experiments, introduction of electron-withdrawing substituents onto the aryl ring and/or the vinyl groups of PPV, is a convenient structural modification to lower both the HOMO and LUMO energy of the polymer, enabling the construction of more efficient devices with relatively high work function metals, such as aluminum [1]. Actually, bilayer devices based on PPV polymers functionalized with strong electron-withdrawing substituents, such as the cyano group, and aluminum electrode displayed efficiencies as high as 4% [2,3,4,5]. The strong electronegativity of the fluorine atom suggested its use as a substituent able to lower the HOMO and LUMO energies, thus enhancing the electroluminescence efficiency as in the case of the cyano substituent, and at the same time increasing the stability of the PPV backbone due to the high strength of the carbon – fluorine bond. Moreover, lowered HOMO energy would result in increased oxidation potential and, again, in enhanced stability against photo-oxidation. Functionalization of PPV polymers with fluorine atoms represents also a strategy for tuning the emission color, and in particular to obtain blue-emitting organic materials. While efficient emission over the color spectrum ranging from green to red can be obtained with PPV polymers, blue emitting PPVs are uncommon. Blue shift in the absorption and emission spectra of PPVs has been achieved by introduction of sterically hindering substituents, which reduce the effective conjugation length [6,7]. Functionalization with electron-withdrawing groups has been also considered as a possible approach to widen the HOMO-LUMO gap, thus blue-shifting the emission [8,9,10].

The aforesaid considerations prompted us to extensively investigate the synthesis of PPVs selectively fluorinated in different positions of the main chain. Here we report an overview of our work on this subject, covering both the synthetic aspects and the systematic investigation of the dependence of the fluorinated PPVs’ properties on the number and position of the fluorine atoms on the conjugated backbone.

## 2. Synthesis

**Scheme 1:**
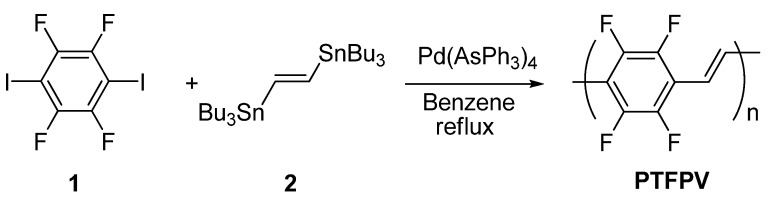
Synthesis of **PTFPV**.

Our approach to the synthesis of PPV polymers is based on the Stille cross-coupling reaction between bis-stannylated vinylic monomers and aryl bis-iodides [11]. In previous work, we extensively investigated the application of this Pd-catalyzed cross-coupling reaction to the synthesis of several alkoxy-substituted PPV polymers [12]. Our investigation on fluorine-substituted poly(*p*-phenylenevinylene)s started with the synthesis of a PPV polymer with fully fluorinated aromatic rings, namely poly(*p*-tetrafluorophenylenevinylene) **PTFPV**. Before our work, the synthesis of this polymer had been unsuccessfully attempted by a soluble precursor route [13] and by self-polycondensation of (*E*)-2-(pentafluorophenyl)ethenyllithium [14]. In our hands, the **PTFPV** was obtained starting from 1,4-diiodotetrafluorobenzene **1** and (*E*)-1,2-bis(tributylstannyl)ethene **2**, which were reacted in the presence of Pd(AsPh_3_)_4_ as the catalyst (generated *in situ* from Pd_2_(dba)_3_ and AsPh_3_), in benzene solution (Scheme 1) [15].

**PTFPV** was recovered from the reaction mixture as an insoluble yellow powder and characterized by Fourier transformed infrared spectroscopy (FTIR), matrix assisted laser desorption ionization-time of flight (MALDI-TOF) mass spectrometry and X-ray photoelectron spectroscopy (XPS). In spite of the complete insolubility of **PTFPV** in all common organic solvents, we were able to record its mass spectrum by MALDI-TOF technique, using a protocol previously described for insoluble polyamides [16]. Thus, we reported the first mass spectrum of an insoluble conjugated polymer [15]. In the MALDI-TOF analysis, peaks with masses ranging from about 500 to 4500 amu were recorded, and molecular weights Mw = 2400 and Mn = 1700, with a polydispersity index *D* > 1.4, were determined. However, mass discrimination phenomena at the higher molecular weight end, which are intrinsic in the MALDI-TOF analysis, can lead to an underestimate of the real molecular weight. From XPS characterization, assuming statistical chain terminations with one iodine atom and one tributylstannyl group, an average polymerization degree of approximately 17–20 arylenevinylene units could be calculated. 

**Scheme 2:**
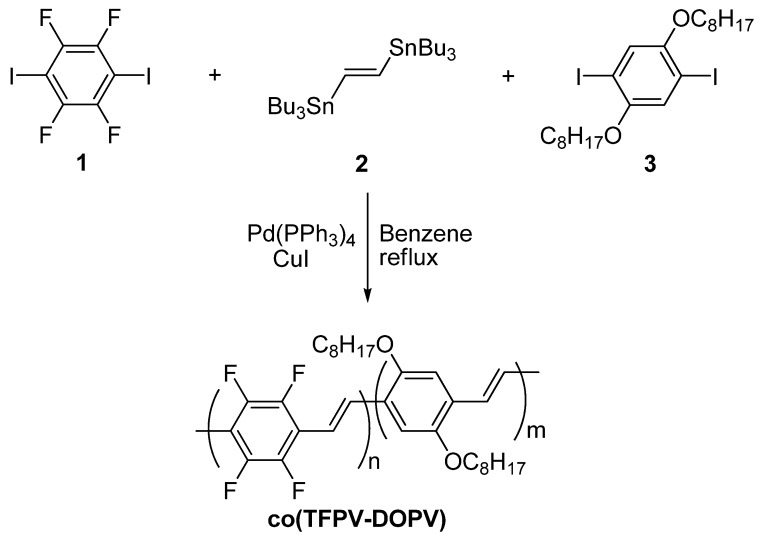
Synthesis of **co(TFPV-DOPV**).

The synthetic investigation was then extended to a random copolymer of 2,3,5,6-tetrafluorophenylenevinylene and 2,5 dialkoxyphenylenevinylene **co(TFPV-DOPV)** [17,18,19]. The synthesis of **co(TFPV-DOPV)** was based on the Stille cross-coupling reaction between (*E*)-1,2-bis(tributylstannyl)ethene **2** and an equimolar amount of the two aromatic monomers 1,4-diiodo-2,3,5,6-tetrafluorobenzene **1** and 1,4-diiodo-2,5-bis(octyloxy)benzene **3**, with tetrakis-(triphenylphosphine) palladium(0) as the catalyst in the presence of copper iodide (Scheme 2) [18].

**Scheme 3:**
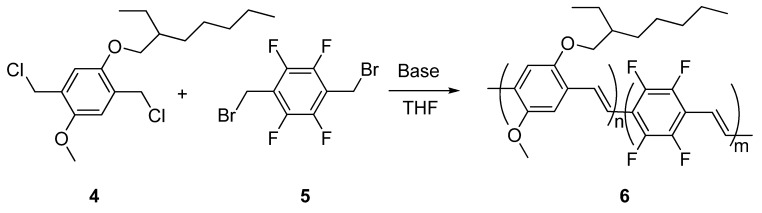
Synthesis of copolymers by Gilch-type polymerization.

It is worth mentioning that, before our work, similar random copolymers **6** were reported by Cacialli *et al.* [20], obtained via a Gilch-type polymerization of bis(halomethyl)phenylenes in THF (Scheme 3). Using different ratios of alkoxy-substituted **4** and fluorinated **5** monomers, various copolymers **6** were obtained incorporating fluorinated units in 7, 14 and 19% weight ratios. The synthesis of polymers incorporating a higher percentage of the fluorinated units was prevented by the loss of solubility due to the reduced number of alkoxy-substituted aromatic rings. 

On the contrary, the **co(TFPV-DOPV)** obtained by our methodology is rich in fluorinated units (>60%) and we attribute this result to its better solubility due to the low molecular weight (Mn = 2200, Mw = 2500, determined by MALDI-TOF mass analysis; Mn = 2200, Mw = 3400, determined by size exclusion chromatography SEC).

In order to enlarge the series of possible structures of fluorinated PPVs, we extended our investigation to polymers with fluorinated double bonds.

Introduction of fluorine atoms on the vinylene units of PPV polymers is synthetically more demanding than the functionalization of the aromatic rings. As a matter of fact, before our work, only two reports had been published dealing with the synthesis of PPVs with fluorinated vinylene units [21,22]. 

As stated above, the use of PPVs in optoelectronic devices for commercial applications is mainly affected by their poor stability against photodegradation processes, which are primarily localized on the double bonds. In this respect, substitution of C-H bonds in the vinylene units with the stronger and less reactive C-F bonds is expected to increase the stability of the resulting PPVs. In 2004, Suh and co-workers first reported the synthesis of two PPV polymers bearing fluorinated vinylene units via the Gilch reaction [21]. Their protocol requires the use of strong bases, thus its application is limited to monomers that can tolerate such reaction conditions. Moreover, incomplete elimination of halogen atoms and lack of regioselectivity (presence of head-to-head and tail-to-tail couplings besides head-to- tail ones), which are common drawbacks of the Gilch approach [23], can lead to structural defects that disturb the conjugation and thus the optical and electrical properties of the polymers. 

**Scheme 4:**
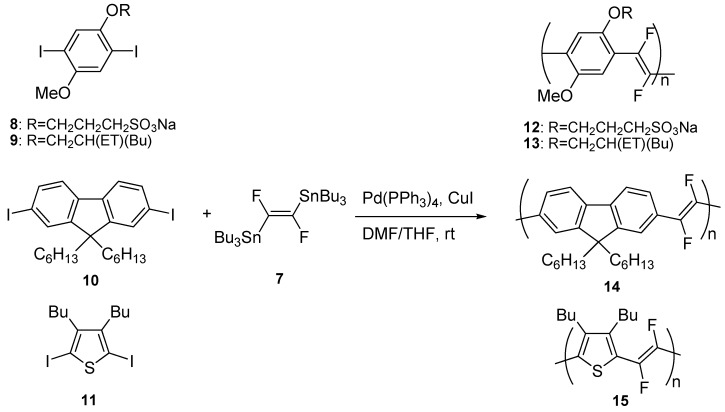
Synthesis of poly(arylenevinylene)s with fluorinated double bonds **12**–**15.**

Our approach to the synthesis of PPV polymers with fluorinated double bonds was again based on the Stille reaction, involving (*E*)-(1,2-difluoro-1,2-ethenediyl)bis(tributilstannane) **7**, synthesized for the first time by Burton *et al.* [24] as the organometallic vinylene monomer. We prepared the compound **7** by a modification of the literature procedure [25] and then we investigated its reactivity with a series of diiodoaryl derivatives, such as 1,4-diiodobenzenes **8** and **9** bearing alkoxy substituents in the 2- and 5-positions, 9,9-dialkylfluorene diiodide **10** and 2,5-diiodothiophene **11** bearing two alkyl groups in the 2- and 3-positions (Scheme 4).

All reactions were carried out using Pd(PPh_3_)_4_ as the catalyst, in the presence of a stoichiometric amount of CuI, a THF/DMF mixture as the solvent, at room temperature for one week reaction time. Polymer **12** was purified by several crystallizations from DMF/acetone and obtained as a yellow-green powder. Polymers **13 (MEH-PPDFV)** and **14** were purified by washing the crude product in a Soxhlet apparatus with hexane (24 h) and ethanol (24 h), then extracting with chloroform, and were isolated as green powders. Finally, polymer **15** was purified by extraction in a Soxhlet apparatus with dichloromethane, followed by precipitation with methanol. The pure product was obtained as a red powder after recrystallization from dichloromethane/methanol. Summing up, the synthetic protocol developed was used to prepare not only PPVs with fluorinated vinylene units and functionalized with different alkoxy substituents on the aromatic rings (**12** and **13**), but also other poly(arylenevinylene)s such as poly(fluorenedifluorovinylene) (**14**) and poly(thienylenedifluorovinylene) (**15**). 

Particularly interesting to note, in view of a systematic investigation of the properties of the fluorinated PPVs in comparison with those of the non-fluorinated counterparts, is the polymer **13** or **MEH-PPDFV**. Actually, **MEH-PPDFV** is a fluorinated analog of **MEH-PPV**, one of the most widely used red-emitting PPV derivatives in electroluminescent devices. Polymer **12** is a fluorinated polyelectrolyte derivative of PPV, which is potentially useful in light emitting electrochemical cells (LECs).

**Table 1:** Yields, molecular weights and polydispersivities of polymers **12**-**15**.

Polymer	Yield (%)	Mw	Mn	Mw/Mn
**12**	55	65,150	40,720	1.6
**13**	87	47,000	27,820	1.7
**14**	82	45,600	23,000	2.0
**15**	50	47,100	16,500	2.9

Average molecular weights Mw and Mn of polymer **12** were determined by a multiangle light-scattering (MALS) detector on-line to a SEC system, while conventional size exclusion chromatography (SEC) was used for **MEH-PPDFV 13**, **14** and **15**. All the polymers show high molecular weights ranging from about 65,000 to 46,000 - an unusual result for PPV polymers obtained by the Stille reaction [12,26,27]. We ascribed the high degree of polymerization observed when the difluoro-stannyl derivative **7** is used as the organometallic vinylene monomer to its better reactivity with respect to the corresponding non-fluorinated vinylene bis-organostannane **2**. 

**Scheme 5:**
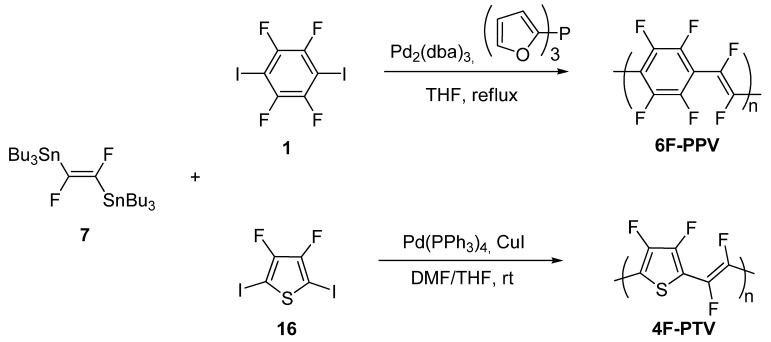
Synthesis of the perfluorinated poly(arylenevinylene)s **6F-PPV** and **4F-PTV**.

More recently, we have extended the Stille polymerization to the preparation of the first two fully fluorinated poly(arylenevinylene)s, namely poly(1,4-tetrafluorophenylenedifluorovinylene) **6F-PPV** and poly(2,5-difluorothienylenedifluorovinylene) **4F-PTV** [28]. Polymers **6F-PPV** and **4F-PTV** were synthesized by reacting **7** with the corresponding bis-iodo perfluorinated aromatic monomers **1** and **16**, respectively, as shown in Scheme 5. 

The effective catalyst for the cross-coupling of (*E*)-(1,2-difluoro-1,2-ethenediyl)bis(tributylstannane) **7** with 1,4-diiodotetrafluorobenzene **1**, was generated *in situ* from Pd_2_(dba)_3_ and tri(2-furyl)phosphine, and the reaction mixture was refluxed for one week. Polymer **4F-PTV** was synthesized by reacting 3,4-difluoro-2,5-diiodothiophene **16** with (*E*)-(1,2-difluoro-1,2-ethenediyl)bis(tributylstannane) **7** using Pd(PPh_3_)_4_ as the catalyst in the presence of a stoichiometric amount of CuI, in a DMF/THF mixture as the solvent. 

## 3. Spectroscopic Properties

**Figure 1:**
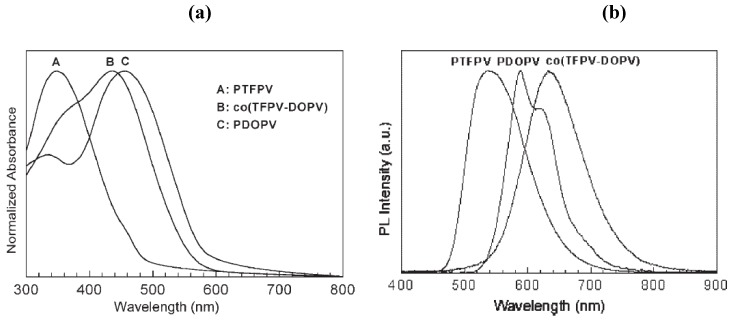
**(a)** UV-vis absorption spectra of **PTFPV**, **PDOPV**, and **co(TFPV-DOPV)** thin films measured at room temperature. **(b)** PL spectra of thin films of **PTFPV**, **PDOPV**, and **co(TFPV-DOPV)** measured at room temperature. Reproduced with permission from ref. 18. Copyright Wiley-VCH Verlag GmbH & Co.KGaA.

**Figure 2:**
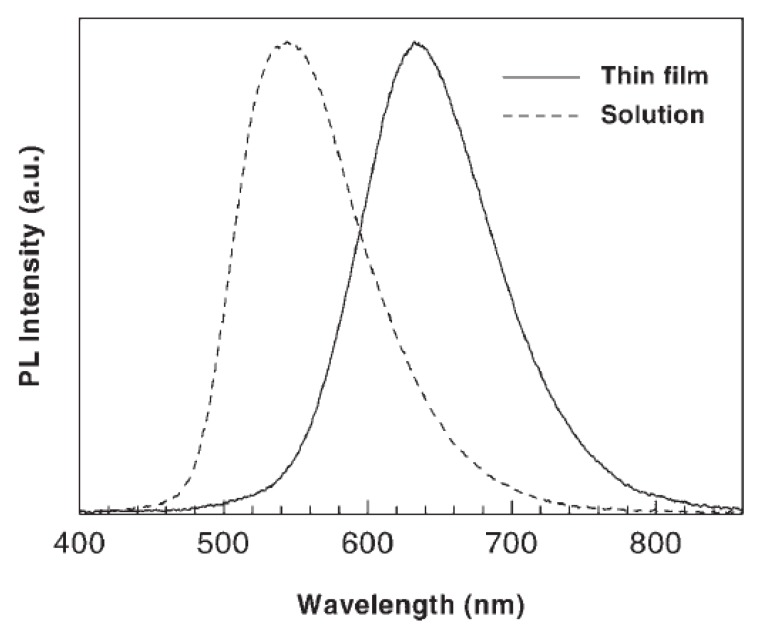
PL spectra of **co(TFPV-DOPV)** in solution (dotted line) and as a thin film (solid line) measured at room temperature. Reproduced with permission from ref. 18. Copyright Wiley-VCH Verlag GmbH & Co.KGaA.

The UV-vis absorption spectra in Figure 1a and the photoluminescence (PL) spectra in Figure 1b show a comparison of the spectroscopic properties of the **PTFPV** with those of the alkoxy substituted polymer **PDOPV** and of the copolymer **co(TFPV-DOPV)** measured in thin film. Films of **PDOPV** and **co(TFPV-DOPV)** were deposited via spin casting from chloroform solution, whereas the **PTFPV** thin film was thermally evaporated under high vacuum (10^-6^ mbar), because this polymer is insoluble in common organic solvents. The fundamental absorption bands of **PDOPV** and **PTFPV** (λ_max_ = 460 nm and λ_max_ = 350 nm, respectively) in Figure 1a are assigned to the transition between the highest occupied and the lowest unoccupied molecular orbitals delocalized along the polymer backbone (HOMO-LUMO transition). The blue shift observed for **PTFPV** compared to **PDOPV** can be attributed to the inductive electron-withdrawing effect of the fluorine atoms on the aromatic rings of the conjugated backbone [29], which is opposite to the electron donating effect of the alkoxy substituents on the **PDOPV** main chain. The origin of the weaker band at shorter wavelengths (λ = 335 nm) in the spectrum of **PDOPV** can be ascribed to additional transitions between orbitals. These transitions are dipole forbidden in unsubstituted PPV, but can be observed in dialkoxy PPV derivatives, due to symmetry breaking of the charge distribution induced by the substituents [30]. The absorption spectrum of the copolymer **co(TFPV-DOPV)** shows two resonances at λ = 440 nm and λ = 360 nm, which are close in energy to the main absorption bands of the parent homopolymers. This structure of the spectrum suggests the presence of two differently substituted phenylenevinylene segments, randomly arranged in the copolymer. The electronic transition at 440 nm originates from chain segments containing the alkoxy substituted monomers and it is slightly blue-shifted compared to the transition observed in **PDOPV**, due to the close presence of electron-poor **PTFPV** segments. On the contrary, the resonance at 360 nm is red-shifted with respect to the corresponding transition in **PTFPV**, because of the presence of adjacent **PDOPV** segments with donor substituents. The PL spectra (Figure 1b) of thin films of the investigated polymers are all significantly red-shifted with respect to the corresponding absorption spectra. The PL spectra of **PDOPV** and **co(TFPV-DOPV)**, in particular, show Stokes’ shifts of 110 and 190 nm, respectively. The large value of the Stokes’ shift of the copolymer **co(TFPV-DOPV)** compared with that of the parent **PDOPV** homopolymer can be attributed to the more effective formation of interchain species in the copolymer in the solid state, due to the simultaneous presence of electron deficient **TFPV** and electron rich **PDOPV** segments [15,31]. This attribution is supported by the broader and structureless shape of the **co(TFPV-DOPV)** PL spectrum in thin film. The presence of exciton interchain migration is confirmed by a comparison of the PL spectra obtained in solution and in thin film of **co(TFPV-DOPV)** (Figure 2). As expected, the light emission from the thin film is strongly red-shifted with respect to that of the solution, due to the enhanced interchain migration of the excitons in the solid state. 

**Figure 3:**
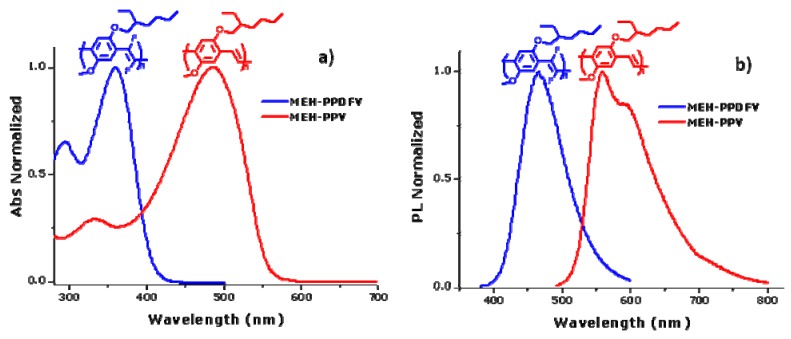
**(a)** Absorption spectra in solution of CHCl_3_ of **MEH-PPV** and **MEH-PPDFV**. **(b)** Emission spectra in solution of CHCl_3_ of **MEH-PPV** and **MEH-PPDFV**.

The comparison of the absorption and emission spectra of **MEH-PPV** with that of **MEH-PPDFV** in solution shows the effect of the introduction of fluorine atoms on the double bonds of alkoxy-substituted PPV polymers. A blue shift of 120 nm of the absorption maximum in chloroform solution is measured for the polymer with the fluorinated double bonds **MEH-PPDFV** compared with the non fluorinated **MEH-PPV**, and a corresponding 89 nm blue shift is observed in the PL spectra in solution (Figure 3).

Based on theoretical modeling and spectroscopic measurements of oligomeric model systems, the observed increase in absorption energy can be attributed to a steric effect rather than to the electron-withdrawing character of the fluorine atoms on the double bonds [32]. Actually, using the Time Dependent Density Functional Theory (TD-DFT), the torsional angle between each alkoxy-substituted aromatic ring and the adjacent fluorinated vinylene moiety is calculated to be about 45°-53°, while in the case of the non-fluorinated polymer it is estimated to be between 0° and 20°. The higher torsional angle for the fluorinated polymer can be attributed to repulsive interactions between the fluorine atoms on the double bonds and the oxygen atoms of the neighboring phenylene rings. Such distortion shortens the effective conjugation length of the fluorinated PPV compared to the non-fluorinated analog, thus increasing the band gap. 

Solid state optical properties of **MEH-PPV** and **MEH-PPDFV** have been comparatively investigated by spectroscopic ellipsometry [33]. Thin films of **MEH-PPDFV** have a fundamental maximum absorption up to about 3.7 eV (~ 330 nm), which is the most “blue” value reported so far for a poly(*p*-phenylenevinylene) polymer. More interestingly, the measured maximum absorption in thin film is up to 30 nm blue shifted from the value obtained for the same polymer in chloroform solution (maximum of absorption at 360 nm) [21]. This contradicts the most common observation of a red-shift of the absorption and emission spectra of conjugated polymers in thin film compared with the same spectra measured in solution [34]. This effect may be attributed to a kind of H-aggregation of localized excitons caused by the twisted conformation of the polymer chains [35]. 

**Figure 4:**
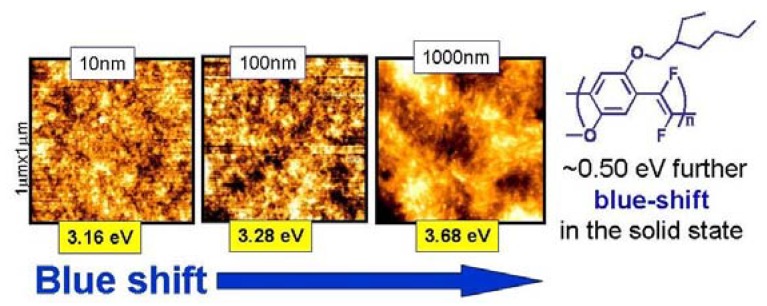
AFM topography of **MEH-PPDFV** films with different thickness and the associated energy gap values measured by spectroscopic ellipsometry.

The optical properties in the solid state have been correlated with the morphology, assembly and supramolecular structure of the thin film that, in turn, can be controlled by the deposition conditions. In particular, it is observed that by increasing from 10 nm to 1000 nm the thickness of thin films obtained by casting from solution a 0.5 eV increase of the energy gap value measured by the spectroscopic ellipsometry technique can be achieved, which is also associated with a change in the morphology of the films, as shown in Figure 4.

**Figure 5:**
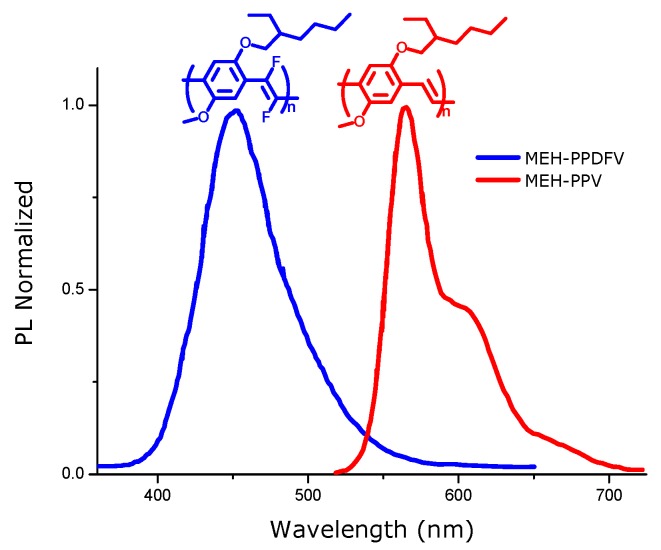
PL spectra of thin films of **MEH-PPV** and **MEH-PPDFV**.

A value of the energy gap as high as 3.68 eV is measured in the solid state for the thickest film obtained. The above **MEH-PPDFV** polymeric film shows strong blue photoluminescence (PL) at room temperature, with a maximum at 458 nm, which is slightly blue-shifted also with respect to the photoluminescence of the polymer in chloroform solution (PL λmax = 468 nm) [25], contrarily to the red-shift of the emission spectrum of **MEH-PPV** from solution to thin film. This blue-shift of the emission in the solid state with respect to the solution indicates that no strong inter-chain aggregation occurs for the **MEH-PPDFV** in thin films. Moreover, the PL λmax of the **MEH-PPDFV** thin film is blue-shifted of about 110 nm compared to that of similar thin film of **MEH-PPV** obtained by the same synthetic methodology (λmax = 568 nm) (Figure 5) and this represents the most blue shifted emission reported so far for a poly(*p*-phenylenevinylene) polymer. 

Finally, the optical properties of the fully fluorinated PPV polymer **6F-PPV** have been investigated. The energy gap measured by spectroscopic ellipsometry (3.34 eV) is strongly blue shifted in comparison with the value measured by the same technique for the corresponding non-fluorinated counterpart (PPV energy gap = 2.4 eV). Several structural factors can be responsible for this strong blue-shift, including the reduced polymer chains length, the electron-withdrawing effect of the fluorine atoms and the steric repulsion between the fluorine atoms *ortho* to the vinylene units on the aromatic rings and those on the double bonds.

## 4. Electroluminescence

**Figure 6:**
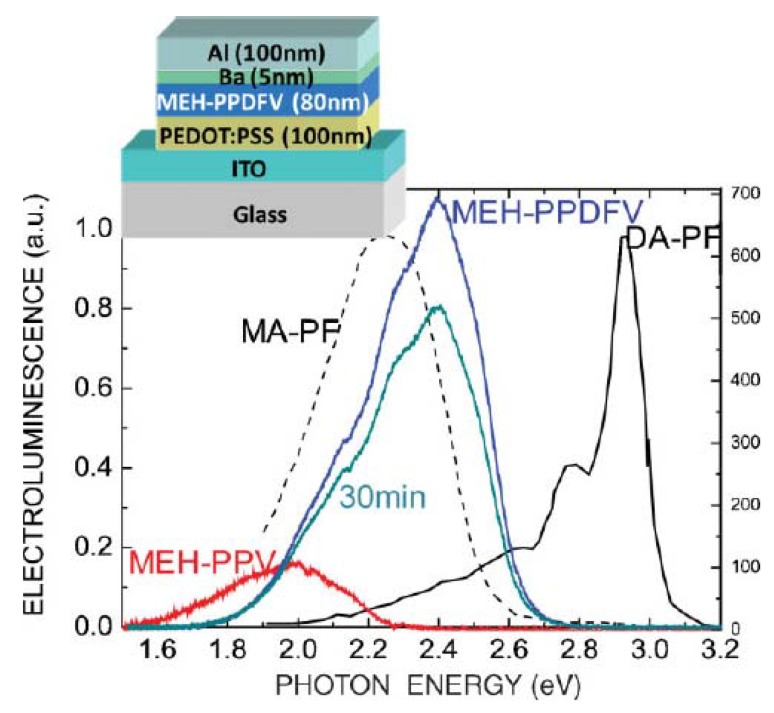
EL spectra in thin films of **MEH-PPV** (red curve) and its fluorinated analog**MEH-PPDFV** at starting time (blue curve) and after 30 min (dark green curve) of continuous operation. For comparison, the normalized EL spectra of an ITO/**MA-PF**/Al and of an ITO/**DA-PF**/Al [37] (left vertical axis) are also shown, where **MA-PF** indicates a 9-monoalkylated polyfluorene and **DA-PF** indicates a 9,9-dialkyl polyfluorene. Reproduced with permission from ref. 33. Copyright Wiley-VCH Verlag GmbH & Co. KGaA.

The EL spectra of **MEH-PPDFV** is blue-shifted with respect to that of **MEH-PPV**, as shown in Figure 6, which compares the EL spectrum obtained from a ITO/PEDOT:PSS/MEH-PPDFV/Ba/Al (ITO: indium tin oxide; PEDOT: poly(3,4-ethylenedioxythiophene); PSS: poly(styrene sulfonic acid)) device with that obtained from a device with the same structure but using **MEH-PPV** as the emissive layer. Both spectra were recorded under the same experimental conditions. The EL spectra of **MEH-PPDFV** and **MEH-PPV** exhibit maxima at approximately 2.4 (*circa* 520 nm) and 1.98 eV (*circa* 625 nm), respectively, corresponding to greenish-blue and red light. The fluorinated **MEH-PPDFV** shows a 100 nm blue-shift compared to the non fluorinated **MEH-PPV**. The strong increase in the EL intensity of the **MEH-PPDFV** compared to the non-fluorinated **MEH-PPV** is also remarkable, and may be correlated to a higher degree of twisting in the conjugated backbone, caused by the F atoms, in agreement with theoretical predictions [32], which should reduce the interchain interactions in the solid state, thus increasing the electroluminescence efficiency.

It is worth noting that **MEH-PPDFV** shows a blue-shift in the absorption and comparable PL emission wavelengths with respect to poly(9,9-dioctylfluorene) **DA-PF,** which is considered one of the most representative blue emitting conjugated polymers exhibiting a π–π* transition peak at 3.23 eV (384 nm) and a PL maximum ranging between 2.85 (436 nm) [36] and 2.7 eV (460 nm) [37]. On the contrary, the blue-greenish electroluminescent peak of **MEH-PPDFV** at 2.4 eV is red shifted with respect to that of **DA-PF** at 2.9 eV, but still blue-shifted with respect to that of the 9-monoalkylated polyfluorene poly(9-octylfluorene) (**MA-PF)** at 2.25 eV [38] (Figure 6).The electroluminescence of **MEH-PPDFV** appears quite stable, since after 30 min of continuous operation in air only a slight decrease in intensity with no red-shift was observed.

## 5. Conclusions

A systematic investigation of the optical properties of poly(*p*-phenylenevinylene)s selectively functionalized with fluorine atoms has been made possible by the development of a general and convenient synthetic methodology based on the Pd-catalyzed Stille cross-coupling reaction between bis(tributyl)stannyl vinylene monomers and functionalized 1,4-diiodoarenes. Changing the monomers and slightly modifying the experimental reaction protocol, it was possible to obtain PPV polymers with fluorine atoms on the aromatic rings and/or on the double bonds, as well as several other functionalized fluorinated arylenevinylene polymers. 

Our study on the impact of the fluorination on optical and electrooptical properties of PPVs reveals that electronic and steric effects caused by the fluorine atoms bonded to the main polymeric backbone deeply affect the characteristics of the conjugated system. In particular, a strong, and somehow unexpected, blue shift in the optical properties of PPVs is induced by the simultaneous presence of fluorine atoms on the double bonds and alkoxy substituents on the aromatic rings. Such structural modification of PPV results in a new kind of blue emitting conjugated polymers.

All the results discussed demonstrate that fluorination is a versatile functionalization that enables to modulate and improve properties of poly(phenylenevinylene) and, more in general, of poly(arylenevinylene) polymers.
